# Molecular Basis of Increased Serum Resistance among Pulmonary Isolates of Non-typeable *Haemophilus influenzae*

**DOI:** 10.1371/journal.ppat.1001247

**Published:** 2011-01-06

**Authors:** Shigeki Nakamura, Mikhail Shchepetov, Ankur B. Dalia, Sarah E. Clark, Timothy F. Murphy, Sanjay Sethi, Janet R. Gilsdorf, Arnold L. Smith, Jeffery N. Weiser

**Affiliations:** 1 Department of Microbiology, University of Pennsylvania, Philadelphia, Pennsylvania, United States of America; 2 Department of Molecular Microbiology and Immunology, Nagasaki University Graduate School of Biomedical Sciences, Nagasaki, Japan; 3 Department of Medicine, University at Buffalo, State University of New York, Buffalo, New York, United States of America; 4 Veterans Affairs Western New York Healthcare System, Buffalo, New York, United States of America; 5 Departments of Epidemiology, Pediatrics and Communicable Diseases, University of Michigan, Ann Arbor, Michigan, United States of America; 6 Center for Childhood Infections, Seattle Children's Hospital Research Institute, Seattle, Washington, United States of America; Fred Hutchinson Cancer Research Center, United States of America

## Abstract

Non-typeable *Haemophilus influenzae* (NTHi), a common commensal of the human pharynx, is also an opportunistic pathogen if it becomes established in the lower respiratory tract (LRT). In comparison to colonizing isolates from the upper airway, LRT isolates, especially those associated with exacerbations of chronic obstructive pulmonary disease, have increased resistance to the complement- and antibody-dependent, bactericidal effect of serum. To define the molecular basis of this resistance, mutants constructed in a serum resistant strain using the *mariner* transposon were screened for loss of survival in normal human serum. The loci required for serum resistance contribute to the structure of the exposed surface of the bacterial outer membrane. These included loci involved in biosynthesis of the oligosaccharide component of lipooligosaccharide (LOS), and *vacJ*, which functions with an ABC transporter encoded by *yrb* genes in retrograde trafficking of phospholipids from the outer to inner leaflet of the cell envelope. Mutations in *vacJ* and *yrb* genes reduced the stability of the outer membrane and were associated with increased cell surface hyrophobicity and phospholipid content. Loss of serum resistance in *vacJ* and *yrb* mutants correlated with increased binding of natural immunoglobulin M in serum as well as anti-oligosaccharide mAbs. Expression of *vacJ* and the *yrb* genes was positively correlated with serum resistance among clinical isolates. Our findings suggest that NTHi adapts to inflammation encountered during infection of the LRT by modulation of its outer leaflet through increased expression of *vacJ* and *yrb* genes to minimize recognition by bactericidal anti-oligosaccharide antibodies.

## Author Summary

*Haemophilus influenzae* generally colonizes the human upper respiratory tract. When isolated from the lower respiratory tract, this opportunistic pathogen is associated with inflammatory conditions such as pneumonia and exacerbations of chronic obstructive pulmonary disease (COPD). Here we show that one of the adaptations made by *H. influenzae* isolated from the lower respiratory tract is increased resistance to the bactericidal effect of antibody and complement. To define the mechanism for increased resistance, mutants were screened to identify the complete set of genes required to inhibit killing by antibody and complement. These included multiple genes that all contribute to biosynthesis of the organism's surface oligosaccharide (lipooligosaccharide), which is targeted by bactericidal antibody. Our results also revealed a novel function for additional genes that maintain the lipid asymmetry of the surface membrane and thereby limit recognition of the pathogen by anti-oligosaccharide antibodies.

## Introduction

The mucosal surface of the human nasopharynx is serially colonized by different strains of *Haemophilus influenzae* [1]. When host factors allow this opportunistic pathogen to gain access to the normally sterile parts of the respiratory tract, inflammatory diseases such as otitis media, sinusitis or pneumonia may result [2]. Widespread immunization against encapsulated strains with the type b polysaccharide has greatly reduced the incidence of invasive disease by *H. influenzae* in children. However, non-typeable strains (NTHi), which do not express a capsule, remain amongst the most common etiologic agents of localized infectious diseases of the airway in all age groups [3]. The damaged airways in adults with chronic obstructive pulmonary disease (COPD) are especially susceptible, and identification of a newly acquired NTHi isolate in sputum is temporally associated with exacerbations of disease symptoms and decline in pulmonary function [4], [5]. COPD ranks as the fourth leading cause of death in the US and is rapidly becoming recognized as a public health problem of similar proportions in other parts of the world [6], [7].

Characteristics of the organism that allow it to transition from its commensal state in the upper airway and survive the inflammatory milieu of the lower respiratory tract (LRT) and elsewhere are poorly understood. In particular, during early infection this predominantly extracellular pathogen will be exposed to increasing levels of natural (i.e. pre-existing) antibody and complement produced locally or extravasated from serum. For encapsulated *H. influenzae*, the thick polysaccharide coat protects the organism from recognition by immunoglobulin, the activation of complement and complement-dependent bactericidal activity. For gram-negative bacteria, the exposed surface is its outer membrane, an asymmetric lipid bilayer consisting of an outer leaflet of lipid A attached to a polysaccharide (LPS) and an inner leaflet of phospholipid [8], [9], [10]. For *H. influenzae*, LPS is referred to as a lipooligosaccharide (LOS) because of its more limited number of attached sugars. There is marked strain to strain heterogeneity in the presence and linkages of these sugars and oligosaccharide epitopes these residues generate indicating that antigenic variation may contribute to immune evasion by NTHi [11], [12]. Structural features of the surface oligosaccharide that inhibit complement-dependent killing have been analyzed extensively [13], [14], [15], [16]. The expression of many of these oligosaccharide components is controlled by highly repetitive DNA sequences and, as a consequence of slipped stranded mispairing, the expression of oligosaccharide structures is turned on and off at high frequency [13], [17]. While this would predict that the presence of bactericidal antibody and complement would select for variants with increased resistance, many of these structures decorating the surface oligosaccharide are present on both serum sensitive and resistant isolates. Therefore, our current understanding does not fully account for why only some NTHi are serum resistant and how this phenotype correlates with the pathogenicity of the species.

In this study, we addressed whether increased resistance to the complement-mediated bactericidal activity of normal human serum is a characteristic of isolates from the LRT. We then used a whole genomic approach to identify the genes required for the expression of serum resistance among these isolates. We describe an important role for genes involved in trafficking of phospholipids in evading natural antibody and the expression of serum resistance by NTHi.

## Results

### Lung isolates have increased serum resistance and decreased binding of natural IgM

**Figure 1:**
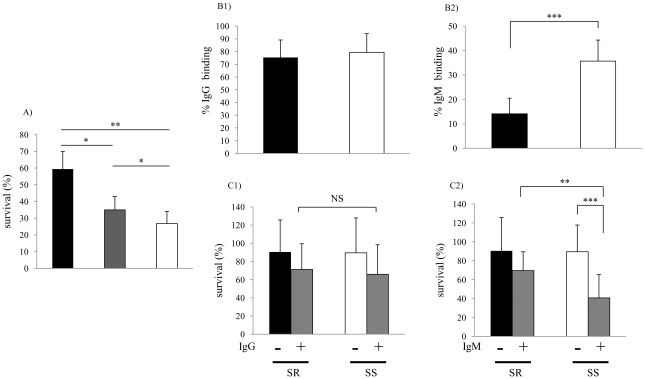
Characterization of clinical isolates. (**A**) Comparison of serum sensitivity between lower and upper respiratory tract isolates. Survival was determined over 60 min in 5% normal human serum and expressed relative to controls in which complement was inactivated. Groups included lower respiratory tract isolates from patients with chronic obstructive pulmonary disease (COPD) at the time of clinical exacerbation (black bars; n = 11); isolates from patients with COPD during clinically stable periods (grey bars; n = 11); and upper respiratory tract colonizing strains (white bars; n = 25). Values are the mean of three determinations in triplicate ± SEM. (**B**) Comparison of antibody binding in serum resistant (>50% survival in pooled NHS, black bars; n = 10) and serum sensitive (<50% survival in pooled NHS, white bars; n = 10) isolates. B1 and B2 show percent of IgG and IgM bound following incubation in 5% heat-inactivated NHS as determined by flow cytometry, respectively. (**C**) Serum IgM contributes to bactericidal killing of clinical isolates of NTHi. Strains were incubated with or without IgG (C1, 0.25 µg/ml) or IgM (C2, 0.07μg/ml) purified from NHS for 60 min in 2.5% baby rabbit serum as a complement source. Percent survival was calculated by viable counts with and without antibody. The mean values of two independent experiments in triplicate are shown ± SD, ^*^ *P*<0.05, ^**^ *P*<0.01, ^***^ *P*<0.001. Serum sensitive (SS), Serum resistant (SR).

Collections of recent clinical isolates maintained with minimal *in vitro* passage were compared for their ability to survive following a 60 min incubation in 5% normal human serum (NHS). Bactericidal activity was complement-dependent, since killing was not observed in controls using heat-inactivated serum. Sputum isolates from the lower respiratory tract (LRT) (n = 22) were significantly more serum resistant than colonizing strains (n = 25) cultured from the upper respiratory tract (Fig. 1A). Among the LRT isolates, those obtained at the time of a COPD exacerbation were the most serum resistant. Next, we examined whether differences in serum resistance correlated with the binding of immunoglobulin present in normal human serum as measured by flow cytometry. There was no difference between serum resistant and serum sensitive isolates in binding of IgG (Fig. 1B1). In contrast, the serum sensitive strains bound significantly more IgM than serum resistant strains (Fig. 1B2). There was no difference between serum resistant and serum sensitive strains in killing by baby rabbit serum (2.5%), which lacks natural antibody to *H. influenzae*, as a source of complement (Fig. 1C). Addition of IgM, but not IgG, purified from NHS to baby rabbit serum significantly enhanced killing of serum sensitive, but not serum resistant isolates (Fig. 1C). Together these results demonstrate 1) an association between serum resistance and the ability of NTHi to infect the LRT and 2) that resistant isolates bind less natural, bactericidal IgM.

### Genetic basis of increased serum resistance

**Figure 2:**
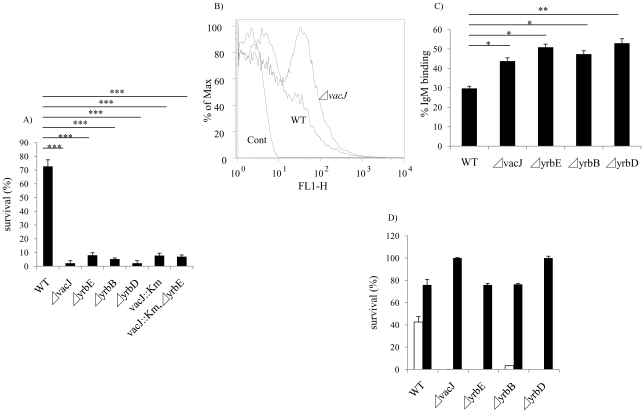
Characterization of *vacJ* and *yrb* mutants. (**A**) Effect of mutations in *vacJ* and genes of the *yrb* ABC transporter on serum resistance of strain R2866. Survival was determined over 60 min in 5% normal human serum and expressed relative to controls in which complement was inactivated. (**B**) Representative histogram comparing the binding, as measured by fluorescence intensity (x-axis), of total IgM purified from normal human serum to parent strain (WT) or *vacJ* by flow cytometry. Control performed without IgM (**C**) Percent IgM binding for each mutant was determined by calculating the percentage of 50,000 events with an increase in mean fluorescence intensity following incubation in 5% heat-inactivated normal human serum compared to no serum controls. (**D**) Survival of mutants in 10% normal human serum in the presence (black bars) or absence (white bars) of Mg-EGTA to inhibit the classical pathway of complement activation. Values represent two independent experiments in triplicate ± SD. ^*^ *P*<0.05, ^**^ *P*<0.01, ^***^ *P*<0.001.

In order to identify the complete set of genes required for serum resistance in NTHi, we screened *mariner* transposon mutants generated in strain R2866, a previously described serum resistant isolate for which the whole genome sequence was available, for increased susceptibility to NHS [18]. A total of 6912 mutants were individually screened to provide ∼4-fold representation of open reading frames. Genomic DNA from candidates showing <10% survival was back transformed into the parent, R2866, and these back transformants were tested to confirm that the insertion mutation conferred a serum sensitive phenotype. Sixty serum sensitive mutants (representing 0.87% of the total strains screened) were identified and for these the *mariner* insertion site was defined. We focused on the genes (13 total and 12 of ‘known’ function) for which there was more than a single ‘hit’(Table 1). Eight loci, including *lgtC, galE, waaQ, lic2A, lex2B, lpsA, yhxB* and *galU*, function in the biosynthesis of the surface oligosaccharide and these were not considered further [19]. The most striking effect on serum resistance (Fig. 2A) was observed with mutations in HI0718 (encoding VacJ, a putative lipoprotein), and in a separate operon with ‘hits’ in HI1083, HI1085, and HI1086 (encoding orthologs of other gram-negative species; YrbB, a putative NTP binding protein; YrbD, an ABC transporter periplasmic protein; and YrbE, an ABC transporter permease, respectively). VacJ, YrbD and YrbE each share homology (>60% sequence identity) with members of the *E. coli* Mla transport system, which have been proposed to function in preventing phospholipid accumulation in the outer leaflet and thereby maintain the lipid asymmetry and the barrier function of the gram-negative outer membrane [20]. A double mutant in *vacJ* and the ABC transporter gene *yrbE* had a similar serum sensitive phenotype to that of each mutant, which confirmed that these may act in the same pathway. The effect on *vacJ* is unlikely to be caused by a polar effect of the insertion mutation, since a mutant in the immediate downstream gene, HI0719, maintained serum resistance (data not shown). There was no effect of *vacJ* or *yrb* genes on LOS or outer membrane protein profiles as assessed following separation using tricine gel electrophoresis followed by silver staining (data not shown).

**Table 1:** List of sites with multiple transposon insertions affecting serum resistance in strain R2866.

HI no. *	Gene name	Protein_id#	Function
0258	*lgtC*	NP_438427*	UDP-galactose-LOS-galactosyltransferase
0351	*galE*	ZP_00156190.2	UDP-glucose 4-epimerase
0461		ZP_00156296.2	Hypothetical protein
0523	*waaQ*	ZP_00349685.1	ADP-heptose-lipooligosaccharide heptosyltransferase III
0550	*lic2A*	ZP_00156370.1	UDP-galactose-LOS-galactosyltransferase
0653	*lex2B*	ZP_00156456.1	UDP-glucose-LOS-glucosyltransferase
0718	*vacJ*	ZP_00156519.2	Lipoprotein (associated to retrograde PLs trafficking)
0740	*yhxB*	ZP_00156601.1	phosphomannomutase
0765	*lpsA*	ZP_00203076.1	LOS-glycosyltransferase
0812	*galU*	ZP_00156667.2	UTP-glucose-1-phosphate uridylyltransferase
1083	*yrbB*	ZP_00156925.2	Putative NTP binding protein
1085	*yrbD*	ZP_00156927.2	ABC transporter periplasmic protein
1086	*yrbE*	ZP_00203123.1	ABC transporter permease

*NCBI Reference Sequence: NC_000907.1 (*Haemophilus influenzae* Rd KW20, complete genome).

# NCBI Reference Sequence: NZ_AADP01000001 and NZ_AADP01000002 (*Haemophilus influenzae* R2866 whole genome).

### vacJ and yrb ABC transporter genes contribute to serum resistance and IgM binding

After incubation in baby rabbit serum, no significant difference was observed in the binding of rabbit complement factor 3 between wild type and *vacJ* mutant (data not shown) indicating a requirement for antibody in the differential susceptibility of the mutants. To determine whether *vacJ* and *yrbE, B* and *D* mediate serum resistance by affecting antibody binding, we compared the deposition of IgG and IgM purified from NHS by flow cytometry. No detectible effect of these mutations on the binding of IgG was observed (data not shown). In contrast, there was a significant increase in the binding of IgM to the mutants (Fig. 2B and 2C). To determine if increased binding of IgM was sufficient to account for loss of serum resistance of the mutants, IgM purified from human serum was used with 2.5% baby rabbit serum as a complement source. Under these conditions each of the mutants was more susceptible to IgM dependent killing (data not shown). To define the complement pathway affected, the survival of the mutants was studied in the presence of Mg-EGTA buffer, which inhibits the classical pathway. When the classical pathway was inhibited, a significant increase in the survival of each mutant was observed (Fig. 2D). The requirement for the classical pathway of complement showed that the anti-bacterial effect of IgM was not caused by agglutination. Together these results demonstrated that *vacJ* and *yrbE, B* and *D* are needed for serum resistance in R2866 by limiting the binding of natural IgM that promotes killing via the classical pathway of complement activation.

### vacJ and yrb ABC transporter genes affect binding of anti-LOS antibody

**Figure 3:**
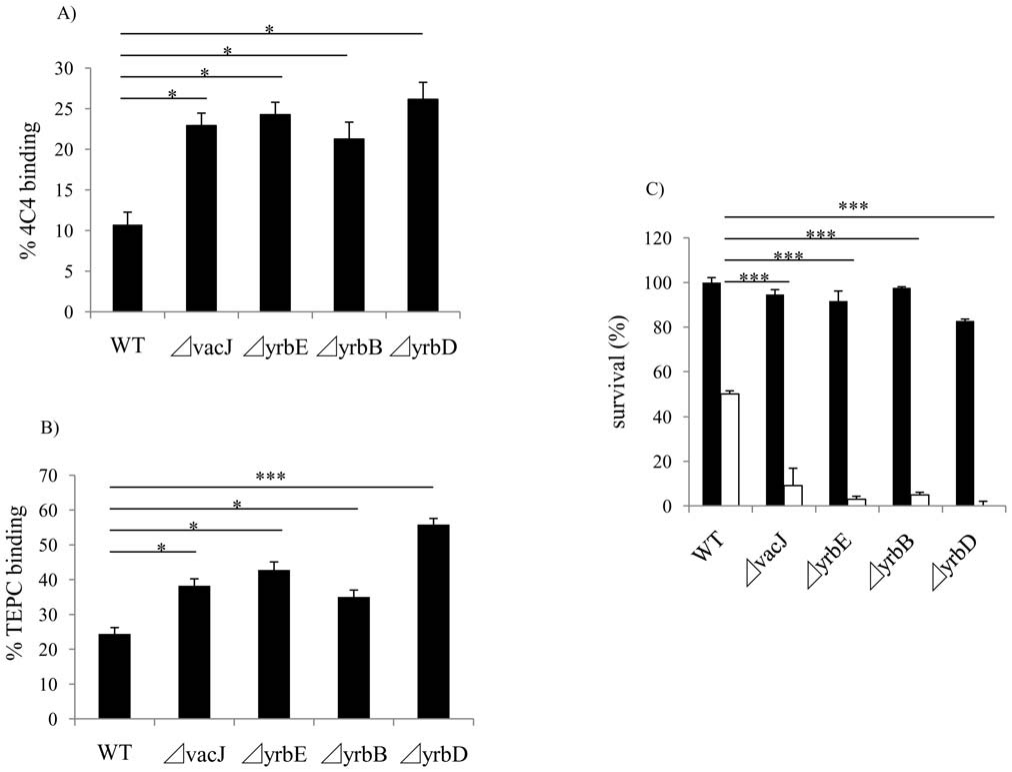
Effect of *vacJ* and *yrb* mutants on antibody binding and bactericidal activity. Binding of (**A**) mAb 4C4 to the LOS structure Galα1-4Gal or (**B**) mAb TEPC-15 to the LOS structure phosphorylcholine was compared by flow cytometry for the mutants indicated. Percent mAb binding for each mutant was determined by calculating the percentage of 50,000 events with an increase in mean fluorescence intensity compared to no primary antibody controls. (**C**) Effect of mutations in *vacJ* and genes of the *yrb* ABC transporter on the bactericidal effect of mAb 4C4. Bactericidal assays were performed with (white bars) or without (black bars) mAb with 5.0% normal mouse serum as a complement source. Values represent two independent experiments in triplicate ± SD. ^*^ *P*<0.05, ^***^ *P*<0.001.

Next, we considered the target of bactericidal, natural IgM affected by mutations in *vacJ* and *yrb* ABC transporter genes. We performed FACS analysis to compare the binding of murine mAbs 4C4 and TEPC-15, which bind specifically to LOS components, Galα1-4Gal and phosphorylcholine, respectively. Since these are both phase variable LOS epitopes, we first enriched for mAb 4C4 or TEPC-15 positive cells by colony immunoblotting. Mutations in *vacJ* and the *yrb* ABC transporter genes significantly increased the binding of mAbs 4C4 and TEPC-15 (Fig. 3A and 3B). Furthermore, mAb 4C4 was bactericidal in the presence of normal mouse serum as a source of complement and each mutant was significantly more sensitive compared to the parent strain. (Fig. 3C). We then investigated whether *vacJ* affects the antibody binding to cell surface proteins by flow cytometry in two different ways. First, we compared binding of the mAb 7B11 to an exposed epitope on outer membrane protein P2 on strain H782 [21]. However, the mutation in *vacJ* in strain H782 did not alter the binding of mAb 7B11 (data not shown). Second, we labeled exposed lysine residues on cell surface proteins by treating whole bacteria with the fluorescent dye Cy5. There was no difference in the levels of bound Cy5 between wild type and *vacJ* mutant (data not shown). Our data suggested that *vacJ* affects binding of antibody to exposed LOS but not outer membrane protein epitopes. Since mAb 4C4 is IgG and TEPC-15 is IgA, the effect on antibody binding and killing was not specific to IgM. This suggests that our observations about natural antibody in NHS could be because the bactericidal antibody targeting LOS is predominantly IgM.

### vacJ and yrb ABC transporter genes affect outer membrane stability

**Figure 4:**
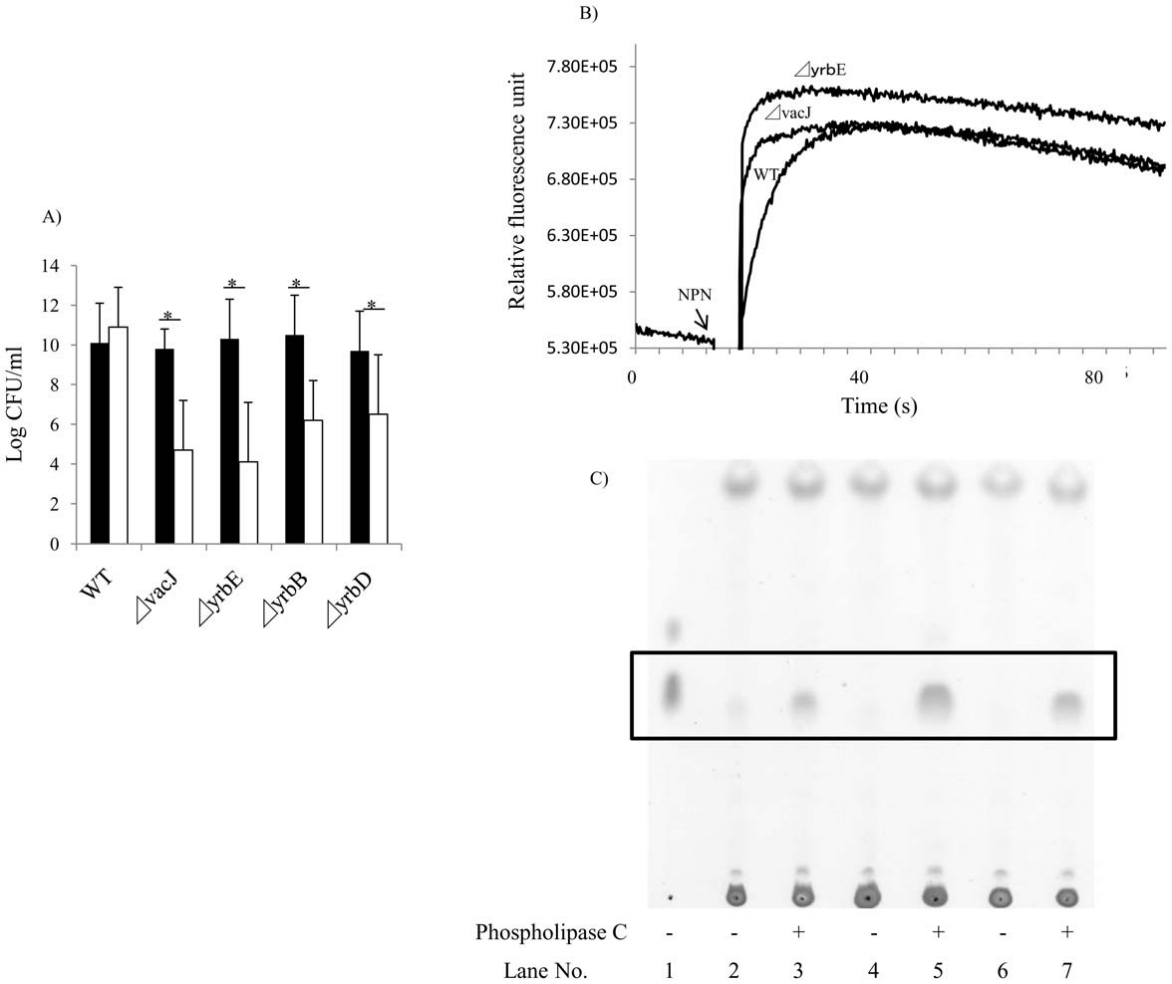
Effect of mutations in *vacJ* and genes of the *yrb* ABC transporter on outer membrane characteristics. (**A**) To compare outer membrane stability, following overnight culture of the strain indicated, viable counts were obtained after incubation at 37°C for 4 h in the presence (white bars) or absence (black bars) of 25 mM EDTA. Values represent two independent experiments in triplicate ± SD. ^*^ *P*<0.05. (**B**) To compare surface hydrophobicity, the rate of uptake of membrane permeant 1-*N*-phenylnaphthylamine (NPN) was monitored by fluorescence. NPN was added at the time indicated and a representative experiment shown. (**C**) To compare amounts of surface phospholipids, diacylglycerol (boxed area) released by phospholipase C treatment of whole bacteria was detected by thin layer chromatography. Lane 1; diacylglycerol (standard), Lane 2 and 3; R2866 (wild type), Lane 4 and 5; 32F2 (*vacJ* mutant), Lane 6 and 7; 69G3 (*yrbE* mutant).

To test whether *vacJ* and *yrb* ABC transporter genes affect the integrity of the outer membrane, we analyzed the sensitivity of the mutants to small antimicrobial compounds, including vancomycin (MW 1449), novobiocin (MW 613), bacitracin (MW 1423), and polymyxin-B (MW 1302). There was no difference in sensitivity to these compounds compared to the parent strain suggesting that the outer membrane barrier of the mutants is largely intact (data not shown). We then addressed the stability of the outer membrane by the addition of EDTA, which chelates divalent cations and compromises the outer leaflet by interrupting intermolecular associations between LOS phosphate groups. A concentration of 25 mM EDTA had no effect on the parent strain, but resulted in a >3-log decrease in viability for each of the mutants (Fig. 4A). Similarly, the mutants were sensitive to the detergent deoxycholate at a concentration 4-fold lower than that required to inhibit growth of the parent strain (data not shown). Our observations of increased sensitivity to EDTA and deoxycholate demonstrated that the stability of the outer membrane was impaired in the mutants. Next, we compared the physical properties of the outer leaflet of the mutants by measuring the rate of uptake of 1-*N*-phenylnaphthylamine (NPN), a probe that changes fluorescence upon transfer from a hydrophilic to hydrophobic environment. We predicted that higher phospholipid content would increase the hydrophobic character of the cell surface of the mutants. As shown in Fig. 4B, *vacJ* and *yrbE* mutants had more rapid NPN uptake than the wild type strain, demonstrating that both mutants have increased surface hydrophobicity. In addition, we directly compared the content of surface exposed phospholipids by treating whole cells with phospholipase C and then detecting released diacylglycerol using thin layer chromatography (Fig. 4C). In comparison to the parent strain, amounts of released diacylglycerol were increased in *vacJ* (spot density increased 219%) and *yrbE* (spot density increased 143%) mutants, showing that the amount of surface phospholipid accessible to phospholipase C treatment was increased in the mutants. The similar results for each mutant provided further evidence that *vacJ* and *yrb* genes act in the same pathway and were consistent with their previously proposed function in *E. coli* in excluding phospholipids from the outer leaflet described [20]. Together our findings suggested that maintaining the asymmetry of the outer leaflet by the exclusion of phospholipids is important in limiting recognition of surface oligosaccharide epitopes by antibody.

### Expression of vacJ and yrb ABC transporter genes correlates with serum resistance in clinical isolates

**Figure 5:**
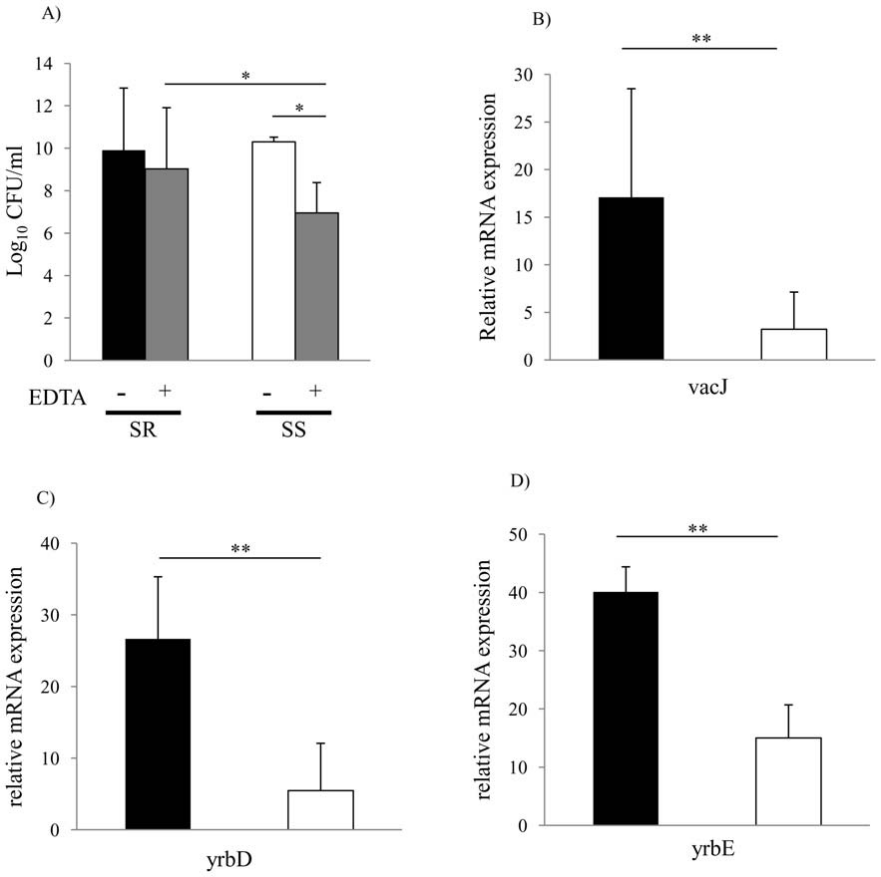
Membrane stability and *vacJ* and *yrb* expression among clinical isolates. (**A**) Outer membrane stability of serum sensitive (n = 31) compared to serum resistant (n = 16) clinical isolates. Following overnight culture, viable counts were obtained after incubation at 37°C for 4 h with or without 25 mM EDTA. Values represent two independent experiments in triplicate ± SD. ^*^ *P*<0.05, Serum sensitive (SS), Serum resistant (SR). (**B**) Relative expression of *vacJ* mRNA, (C) *yrbD* and (D) *yrbE* by qRT-PCR in serum sensitive (white bar, n = 7) and serum resistant isolates (black bar, n = 7). Error bars indicate SD, ^**^ *P*<0.01.

**Figure 6:**
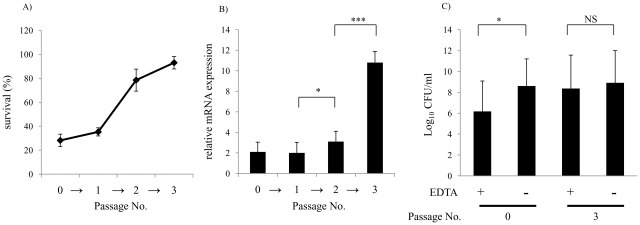
Repeated serum treatment selects for serum resistance, increased *vacJ* expression, and increased outer membrane stability. (**A**) Serum sensitive clinical isolate H725 was treated three times in 2.5% NHS and survival quantified after the passage indicated. (**B**) Following each passage in the bactericidal assay survivors were tested for relative expression of *vacJ* mRNA by qRT-PCR and (**C**) survival in the presence of 25 mM EDTA. Values represent two independent experiments in triplicate ± SD.^*^ *P*<0.05, ^***^ *P*<0.001.

To investigate the relationship between outer leaflet stability and serum resistance in NTHi, we compared the sensitivity of serum resistant and serum sensitive clinical isolates to EDTA. As shown in Fig. 5A, serum resistant isolates were significantly more resistant to EDTA than serum sensitive isolates. We then determined whether differences in serum resistance and resistance to EDTA correlated with the expression of *vacJ* and *yrb* genes by qRT-PCR. As shown in Fig. 5B, *vacJ* expression was ∼5-fold higher in serum resistant strains compared to serum sensitive isolates. In addition, *yrbE* and *yrbD* expression was ∼3-fold higher in serum resistant compared to serum sensitive isolates (Fig. 5C and 5D). To further investigate the relationship between serum resistance and *vacJ* expression, we serially passaged strain H725, a serum sensitive clinical isolate, in 2.5% NHS to select for variants with an increasing capacity to resist the bactericidal effect of serum. With each passage H725 became more serum resistant and this adaptation was associated with a stepwise increase in *vacJ* expression and resistance to EDTA (Fig. 6A–C). Our data suggest that among clinical isolates differences in serum resistance correlate with the level of expression of *vacJ* and its effect on stability of the outer leaflet of NTHi.

## Discussion

Although generally co-existing in a commensal relationship with its host, NTHi is able to survive the robust inflammatory response it induces in normally sterile sites in the respiratory tract such as the lung. Since humans are serially colonized beginning early in childhood, prior exposure to NTHi, as well as to other microbial species that induce cross reactivity, provides an abundant source of natural antibody [22], [23]. Individuals with defects in generating antibody are particularly susceptible to recurrent respiratory tract infection with NTHi [24]. To survive the inflammatory response in the LRT, this pathogen has to evade the effects of the host's pool of pre-existing antibody, which when bound to the bacterial surface activates complement and induces lytic killing. In this report we used a serum killing assay to show that survival of NTHi isolated from the LRT is associated with increased resistance to the complement-dependent bactericidal effect of antibody. Additional evidence in support of this conclusion is the finding that serum resistance was highest among the isolates obtained at the time of COPD exacerbations when clinical signs of inflammation, such as increased production of sputum, are more pronounced. Although antibodies of other isotypes can be bactericidal, we found that most of the natural bactericidal antibody present in NHS is IgM. Our findings demonstrate that serum killing correlates with binding of natural IgM followed by activation of the classical pathway of complement. Observations on the prominence of anti-LOS IgM in the bactericidal activity of NHS correlate with previous reports looking at natural bactericidal antibody present in animal sera [25]. Findings in the current study are also consistent with prior reports from this laboratory using a mouse model of airway infection showing that complement and natural antibody protect the host from NTHi [26].

Previous analysis of serum resistance in NTHi has been limited by the marked heterogeneity within and between strains. This is largely caused by the rapid variation in the expression of surface antigens targeted by natural antibody and demonstrates the ability of the organism to escape antibody-dependent, complement-mediated killing. Several specific oligosaccharide structures that contribute to serum resistance have been described. Some strains express siayltransferases that use serum CMP-NANA to cap the oligosacchaide with sialic acid, which inhibits the activation of complement through the alternative pathway [16]. The disaccharide Galα1-4Gal, which mimics the human P^k^ blood group antigen, blocks recognition by anti-LOS antibodies [13]. The oligosaccharide structures requiring the galactosyltransferase LgtC affects expression of Galα1-4Gal modulating deposition of C4b and activation of the classical pathway [15]. These oligosaccharide decorations are each variably expressed within and between strains. Our finding that 8 of the 13 genes necessary for the expression of serum resistance in a highly serum resistant NTHi strain function in biosynthesis of the surface oligosaccharide highlights its central role in this phenotype and the pathogenicity of the organism. In addition, we demonstrate that the oligosaccharide is the major target of bactericidal human antibody. This may explain the predominance of natural IgM in targeting the NTHi oligosaccharide, since antibody to LPS antigens is dominated by IgM generated by B-1 cells [27]. For example, a large proportion of B-1 cells generate IgM reactive with phosphorylcholine [28].

The selection for a more serum resistant phenotype in the LRT correlated with increased expression of *vacJ* and *yrb* genes, which alter cell surface characteristics and thereby limit the binding of bactericidal antibody. The identification of VacJ and an ABC transporter with related function as necessary for the expression of serum resistance shows that surface characteristics other than structural components of the oligosaccharide contribute to serum resistance of NTHi. The lipoprotein VacJ was previously identified as a virulence determinant contributing to intracellular survival by *Shigella flexneri* [29]. The group of Silhavy proposed that VacJ acts with the Mla ABC transporter to maintain the lipid asymmetry of the outer membrane by recycling phospholipids from the outer leaflet back to the inner leaflet [20]. In *E. coli*, mutants lacking these genes were more sensitive to the presence of SDS plus EDTA added to solid media, but not to antibiotics that need to access targets in the periplasmic space. These observations indicated that 1) stability of the outer leaflet generated through intermolecular bridging of LPS by divalent cations was compromised by the accumulation of phospholipids and 2) the permeability barrier of the outer leaflet remains largely intact in the mutants. We observed similar characteristics of mutants in *vacJ* and Mla ABC transporter homologs in NTHi. In addition, we provided direct evidence that *vacJ* and *yrb* genes function in determining key characteristics of the cell surface (i.e. its hydrophobicity) and that these genes affect amounts of surface exposed phosholipid. The increase in antibody binding and killing seen in these mutants provides a new insight into how these physical characteristics of the outer membrane contribute to serum resistance. Our results suggest that the intramolecular forces bridging LOS molecules also serve to limit binding of antibodies to the oligosaccharide. Thus, when phospholipids are more thoroughly excluded (high expression of *vacJ* and *yrb* genes), LOS molecules are more tightly packed and accessibility of LOS epitopes is restricted (serum resistance). Whereas when phospholipids accumulate in the outer leaflet (low expression of *vacJ* and *yrb* genes), intermolecular associations of LOS molecules are interrupted allowing for increased access of oligosaccharide epitopes recognized by bactericidal antibodies (serum sensitivity).

Interestingly, the expression of *vacJ* and *yrb* genes, which are required for serum resistance, is variable within and between NTHi strains. Serum resistant isolates demonstrated increased levels of transcription compared to serum sensitive isolates and serial passage of a sensitive isolate in serum selected for increased resistance and resulted in a higher level of *vacJ* expression. Thus, differences in transcription of *vacJ* are another factor accounting for the marked differences in serum resistance that characterizes this species. It is somewhat surprising that for many clinical isolates levels of *vacJ* expression is low enough to affect the stability of the outer membrane. This suggests that some level of phospholipid accumulation in the outer leaflet is tolerated by NTHi and implies that there must be an advantage to a less stable, more hydrophobic outer membrane, particularly during colonization when restricting recognition by antibody may be less critical for survival. It appears, however, that this fitness advantage is lost during infection of the lung when increased *vacJ* and *yrb* gene expression is selected for. Because NTHi resides in the respiratory tract where it is not exposed to the detergent effect of bile in the intestine, the physical requirements of its outer membrane may be different from the paradigm described in the classic studies based on enteric bacteria [30].

In conclusion, we show that resistance to the bactericidal effect of immunoglobulin together with complement correlates with the ability of NTHi to infect the human LRT. Analysis of a serum resistant isolate revealed that genes contributing to the biosynthesis of its surface oligosaccharide are required for this phenotype. In addition, we describe a novel mechanism for serum resistance whereby NTHi limits the binding of bactericidal anti-LOS antibody by increasing the exclusion of surface phospholipids.

## Materials and Methods

### Bacterial strains and growth conditions

Strains used in this study are listed in Table 2. *COPD strains.* Strains were isolated from expectorated sputum samples as part of a prospective study at the Buffalo VA Medical Center (4). COPD exacerbation strains fulfilled the following criteria: 1) First isolation of the strain in an adult with COPD based on molecular typing of isolates recovered from monthly sputum cultures; 2) NTHi is the only potential pulmonary pathogen isolated in the sputum sample; 3) simultaneous onset of clinical symptoms of an exacerbation (increased sputum volume, increased sputum purulence and increased shortness of breath compared to baseline symptoms). Non-exacerbation strains were from patients with COPD during clinically stable periods who fulfilled criteria 1 and 2 but symptoms unchanged from baseline upon acquisition of the strain. *Upper respiratory tract strains*. 25 NTHi strains were isolated from throat cultures of 25 healthy children attending 17 different day care centers [31], [32]. Each isolate had a different pulse-field gel electrophoresis pattern and was confirmed to be *H. influenzae* (and not non-hemolytic *H. haemolyticus*) based on previous criteria [1], [33]. The absence of *bexA* and *bexB* confirmed that the isolates were not capsule-negative variants.

**Table 2:** The list of strains, plasmids and primers used in this study.

Strains, plasmids, primers	Description	Reference
Strains		
Rd	Rough type d isolate, genome sequence reference strain	[46]
2019	Clinical isolates of nontypeable *H. influenzae*	[47]
R2866	NTHi clinical isolate from the bloodstream	[18]
32F2	R2866 *vacJ* disrupted by *mariner* Tn	This study
49E3	R2866 *yrbD* ABC transporter periplasmic protein disrupted by *mariner* Tn	This study
66B5	R2866 *yrbB* NTP binding protein disrupted by *mariner* Tn	This study
69G3	R2866 *yrbE* ABC transporter perimease disrupted by *mariner* Tn	This study
H782	Rd transformed with DNA of 2019 to express m7B11 epitope on OMP P2	This study
H725	Clinical isolate of NTHi from lower respiratory tract	This study
H816	H782 transformed with DNA of 32F2 to disrupt *vacJ*	This study
*vacJ*::Km	R2866 transformed with pUCΔ*vacJ*Km^R^ to disrupt *vacJ*	This study
*vacJ*::Km,Δ*yrbE*	69G3 transformed with pUCΔ*vacJ*Km^R^ to disrupt *vacJ*	This study
**Plasmids**		
pEMspec	Contains *mariner* Tn carrying Spec^R^ cassette	[35]
pUCΔ*vacJ*Km^R^	*vacJ* replaced with Km^R^ cassette from pUC4K	This study
**Primers**		
ARB1	GGCCACGCGTGCACTAGTAC (N)_10_ TACNG	[48]
ARB2	GGCCACGCGTGCACTAGTAC	[48]
MAG2F3	GGAATCATTTGAAGGTTGGTA	[49]
MAG2F4	ACTAGCGACGCCATCTATGTG	[49]
Hi_vacJ_H3_F2	AAGCTTAAAATGTAGCAGGTAAACGTCG	This study
Hi_vacJ_R1	ACGTAATGCCATCGTTTTAGAC	This study
Hi_vacJ_B1_F1	CGGGATCCTAAACAGAAAAGTGCGGTAAAAATT	This study
Hi_vacJ_B1_R1	CGGGATCCTTTTAATCCTTACATAAATATGGGATTATTC	This study
Hi_vacJ_F9	CGTGCGTTCTTTAATGACTCTCG	This study
Hi_vacJ_R7	CAATGTGAAGTGGAAAAGCCCC	This study

Strains were routinely grown at 37°C in brain heart infusion broth (Becton Dickinson) supplemented with 2% Fildes enrichment (Remel) and 20 µg/ml β-NAD hydrate (NAD; Sigma). H782 was created from strain Rd by transformation with a PCR product from outer membrane protein P2 locus of strain 2019 followed by screening for expression of the surface epitope recognized by mAb 7B11 epitope [21].

### Ethics statement

The strains from adults with COPD were obtained from subjects enrolled in a study at the Buffalo VA Medical Center that was approved by the IRB of the VA Western NY Healthcare System. All subjects provided written informed consent. Strains collected from healthy children in day care, under protocols reviewed by IRB Health at the University of Michigan, were deemed EXEMPT on the basis of: EXEMPTION #4 (45 CFR 46.101(b)(4)), because the data were collected and analyzed without personal identifiers attached to the bacterial isolates.

### In vitro transposon mutagenesis, DNA transformation and transposon library construction

*Mariner* mutants of strain R2866 were created by *in vitro* transposon mutagenesis as previously described [34]. Briefly, *in vitro* transposition reactions were carried out on NTHi genomic DNA treated with purified MarC9 transposase and *pEMspec* [35]. To repair gaps, reactions were ethanol-precipitated and resuspended in the gap repair buffer [50 mM Tris (pH 7.8)], 10 mM MgCl_2_, 1 mM DTT, 100 nM dNTP, and 50 ng of BSA] and then treated with T4 DNA polymerase (Invitrogen) and *E. coli* DNA ligase (Invitrogen) [36]. DNA was transformed into competent R2866 by the method of Herriott et al [37] and transformants where selected for on sBHI agar (1%) plates containing spectinomycin (100 µg/ml).

### Identification of serum sensitive transposon mutants

To identify NTHi genes essential for serum resistance, *mariner* transposon mutants were screened in a 96-well serum bactericidal assay. Following growth from single colonies in 200 µl sBHI, mutants were diluted to 10^5^ CFU/ml and 10 µl of the culture solution was added to 90 µl consisting of 55 µl PBS, 30μl Hank's buffer (Ca^2+^, Mg^2+^) and 5 µl of normal human serum (NHS). Serum was obtained from a single donor for the initial screen to minimize variability in screening large numbers of mutants. Bacteria were incubated at 37°C for 1 h with shaking before the reaction was stopped at 4°C. Controls included serum from the same donor treated at 56°C for 30 min to inactivate complement. Mutants showing >90% killing in the primary screen were further tested to minimize false positives. Genomic DNA was used to back transform competent R2866 and three spectinomycin resistant colonies were picked and rescreened in the bactericidal assay. The site of the transposon insertion was determined for mutants in which 3/3 back transformants were serum sensitive.

### Arbitrary primed PCR and nucleotide sequence

The primers used for inverse PCR are listed in Table 2. The first round of PCR was performed in a final volume of 50 µl. Primers (0.5 µmol/reaction mixture) ARB1 paired with mag2F3 (transposon) were used in PCR under the following conditions: 1 cycle of 95°C for 8 min; 6 cycles of 95°C for 30 s, 30°C for 30 s, and 72°C for 1.5 min; 30 cycles of 95°C for 30 s, 45°C for 30 s, and 72°C for 2 min; and 72°C for 5 min. A second round of PCR was performed with primers (0.5 µmol/reaction) ARB2 paired with the internal mag2F4 (transposon) primer under the following conditions: 1 cycle of 95°C for 8 min, 30 cycles of 95°C for 45 s, 55°C for 45 s, and 72°C for 1.5 min, followed by 72°C for 10 min. PCR products were purified with the QIAGEN PCR cleanup kit and then used for nucleotide sequence analysis.

### Construction of Δ*vacJ*::Km^R^ mutant

The *vacJ* gene and flanking regions were amplified from strain R2866 using primers Hi_vacJ_H3_F2 and Hi_vacJ_R1 and cloned into pCR2.1TOPO vector. The entire *vacJ* gene was deleted by inverse PCR using Hi_vacJ_B1_F1 and Hi_vacJ_B1_R1 primers introducing a BamHI site. The resulting Δ*vacJ* fragment was subcloned into pUC19 and the kanamycin-resistance cassette from pUC4K was then inserted using BamHI creating pUCΔ*vacJ*::Km^R^. This plasmid was used to transform strains R2866 and 69G3 strains creating single and double Δ*vacJ*::Km^R^ mutants. Disruption of *vacJ* was confirmed by PCR using primers vacJ_ F9 and Hi_vacJ_R7 located outside of the originally cloned region.

### Serum bactericidal assays

To test clinical isolates in bactericidal assays, serum was collected, pooled and stored at -80°C from 5 healthy adult volunteers. Assays were performed with 20 µl of a suspension of midlog phase organisms (OD_620_ 0.3–0.4) diluted to 10^5^ CFU/ml in Hank's buffer with Ca^2+^ and Mg^2+^ (GIBCO, Auckland, New Zealand), 10 µl of NHS, 110 µl of PBS and 60 µl of Hank's buffer with Ca^2+^ and Mg^2+^. After incubation for 60 min at 37°C with rotation, the assay was stopped by cooling to 4°C and dilutions were made for quantitative culture. To inhibit the classical pathway of complement, veronal buffer (pH 7.4) containing 70 mM NaCl, 140 mM glucose, 0.1% gelatin, 7 mM MgCl_2_, and 10 mM Mg-EGTA was substituted for Hank's buffer [38]. To calculate the percent survival, viable counts were compared to controls in which complement activity had been eliminated by heat-inactivation at 56°C for 30 min. IgG was removed from NHS using a protein G column according to the manufacturer's instructions (GE Healthcare, Uppsala, Sweden). The flow-through of IgG column was used for purification of IgM. Purified IgM was obtained by using IgM purification column according to the manufacturer's instructions (GE Healthcare, Uppsala, Sweden). The concentration of purified IgG and IgM were determined by the Micro BCA protein assay (Pierce Chemical Co.) and a titrated enzyme-linked immunosorbent assay (ELISA). Where indicated, three-four week old baby rabbit serum was used as a complement source (Pel-Freez Biologicals, Rogers, AR).

### Colony immunoblotting

Colonies lifted onto nitrocellulose were immunoblotted to separate phase variants as previously described [39]. Briefly, the LOS and ChoP epitope on colonies lifted onto nitrocellulose were detected using mAbs 4C4 against Galα1-4Gal [40] and TEPC-15 (Sigma) against phosphorylcholine followed by alkaline phosphatase–conjugated anti– mouse IgG and IgA, respectively. Single colonies of a uniform phenotype were selected and confirmed by repeated immunoblotting.

### Labeling of surface proteins

200 µl of a suspension of midlog phase organisms, R2866 and *vacJ* mutant, (OD_620_ 0.5) was pelleted, washed twice with 1 ml of 10 mM carbonate buffer (pH 8.5), and then resuspended in 200 µl of buffer (10 mM carbonate, 1 M urea). Surface-exposed lysine residues were labeled with 10 µl of Cy5 (400 pmol, GE Healthcare) on ice in the dark for 20 min [41]. Reactions were stopped by addition of 20 µl of 10 mM lysine. The resulting pellets were washed twice with 500 µl of carbonate buffer, centrifuged, and resuspended in 200μl 1% BSA. Cy5 binding on bacteria was analyzed by flow cytometry with 10,000 events analyzed per sample.

### Deposition of complement factor 3

R2866 and *vacJ* mutant were grown to an OD_620_ ∼0.5. The bacterial suspension (200 µl) was pelleted and resuspended in Hank's buffer with Ca^2+^ and Mg^2+^ (GIBCO, Auckland, New Zealand) with 5% fetal calf serum (HyClone) and incubated with 5μl of baby rabbit sera for 1°h. Bacteria were pelleted and resuspended in Hanks' buffer plus 5% fetal calf serum containing a 1:100 dilution of a FITC-conjugated polyclonal goat anti-rabbit C3 antibody (MP Biomedical Cappel, Irvine, CA) for 60 min at 4°C in dark and analyzed by flow cytometry.

### Quantitative real-time RT-PCR

Total cellular RNA was extracted from mid-log phase grown NTHi clinical isolates by using the RNeasy mini Kit (QIAGEN). To eliminate genomic DNA, samples were incubated with 20 U of RNase-free DNase (QIAGEN) for 20 min at 25°C using the RNeasy columns, according to the manufacturer's instructions. 1 to 1.5 µg RNA was used for reverse transcription in a 20 µl reaction with the high-capacity cDNA reverse transcription kit (Applied Biosystems) together with random primers and 20 U RNase inhibitor (Promega). 1 µl of cDNA from this reaction was used as template with 0.5 µM primers (*vacJ*-F: 5′-TCCGTGGGCATTAGTGAAAT-3′; *vacJ*-R:5′-AATTCTGCATTATTGAGATTTTTCG-3′; *yrbD*-F:5′-TACTGTGACGGCAACTTTCG-3′; *yrbD*-R:5′-AATCGCGATGCTTACTTTCG-3′; *yrbE*-F:5′-TCGTGTTAATCGATTTTTCTGC-3′; *yrbE*-R:5′- CAGGGCCTAATTCTCGTAAAAG-3′) and SYBR Green PCR Master Mix in a 20-μl reaction (Applied Biosystems). Standard runs of the reactions on fast optical 96-well reaction plates (Applied Biosystems) were carried out using the StepOnePlus Real-Time PCR system (Applied Biosystems). The *gyrA* gene (primers *gyr*-F: 5′-GCGTGTTGTGGGTGATGTAA-3′; and *gyr*-R: 5′- GTTGTGCCATACGAACGATG-3′) was used as the internal standard gene for RNA quantity normalization [42]. Quantitative comparison was obtained through the ΔΔ*C*_T_ method as described at http://www3.appliedbiosystems.com/cms/groups/mcb_support/documents/generaldocuments/cms_041435.pdf.

### Antibody binding assays

200 µl of mid-logarithmic-phase bacterial cells (OD_620_ 0.5) were pelleted and resuspended in 200 µl of Hanks' buffer without Ca^2+^ and Mg^2+^ (Gibco) supplemented with 5% fetal calf serum (HyClone). Primary antibodies (1:200 for heat inactivated NHS, 1:50 for mAb 4C4, 1:1000 for mAb TEPC-15, and 1:100 for mAb 7B11) were added to reaction and then incubated at 37°C for 60 min. Bacteria were pelleted and incubated with Hanks' buffer without Ca^2+^ and Mg^2+^ plus 5% fetal calf serum containing a 1:200 dilution of the appropriate secondary antibody; goat anti-human IgG-FITC conjugate (Sigma), goat anti-human IgM FITC conjugate (Sigma), anti-mouse IgG-FITC conjugate (Sigma), anti-mouse IgA-FITC conjugate (Sigma) for 60 min at 4°C in the dark. Reaction mixtures were then washed and resuspended in 200 µl of PBS containing 1% bovine serum albumin and 0.5% paraformaldehyde. A total of 50,000 cells were collected for each sample. All samples were subjected in full volume to flow cytometry analysis on a BD FACS Calibur flow cytometer (BD Biosciences), and groups were compared using FlowJo software (Tree Star).

### Growth inhibition assays

Ethylenediaminetetraacetate (EDTA) was added to 500 µl of an overnight culture in sBHI broth (25 mM final concentration) and incubated at 37°C for 4 h under aeration prior to obtaining viable counts. The minimum inhibitory concentration of vancomycin, novobiocin, bacitracin, polymyxin-B and deoxycholate was determined by broth microdilution method. Briefly, 96-well plates containing 2-fold dilution of chemical were prepared in sBHI and wells were inoculated with ∼5×10^5^ cfu/ml of overnight bacterial cultures. Viable counts were determined after incubation for 18–24 h at 37°C.

### NPN uptake assay

1-*N*-phenylnaphthylamine (NPN) (Sigma) uptake assay was performed as described previously [43]. Briefly, bacteria were grown to OD_620_ 0.5 in sBHI broth. Pellets were washed and resuspended in 5 mmol/L HEPES buffer (pH 7.2). NPN was dissolved in acetone for a 500 µmol/L stock solution and diluted to 40 µmol/L in HEPES buffer. The NPN solution (50 µl) was added after 15 s to the bacterial suspension (150 µl) for a final NPN concentration of 10 µmol/L. Fluorescence was monitored for a total of 90 s using the PTi fluorescence system (Photon Technology International), with excitation at 350 nm, emission at 420 nm, and slit width of 2 nm.

### Phospholipase C treatment

Phospholipase C treatment and thin layer chromatography (TLC) were performed as described previously [44]. 1 L of each bacterial culture were grown in sBHI broth to OD_620_ 0.5 and centrifuged ×5000 g for 20 min. Pellets were washed once and resuspended in a sucrose-PBS-MgCl_2_ buffer [0.4M sucrose, 1×PBS, 15 mM MgCl_2_, (pH 7.5)] to a final volume of 1.8 ml. 200 µl aliquots of bacterial suspension were treated with 5u of phospholipase C from *Bacillus cereus* (Sigma) for 20 min at 37°C. Reactions were stopped by adding 600 µl CHCl_3_-methanol 1:2 (v/v). The lipids were extracted by the method of Bligh and Dyer [45]. Lipid samples were normalized to protein content and separated by TLC on glass backed silica gel 60 plates (Merck) with CHCl_3_-Acetone 94:4 (v/v) solvent system and were stained by iodine. The standard was 10 µg of 1,2-diglycerides from pig liver (Serdary Research Lab.).

### Statistical analysis

All data were analyzed using StatView software (Abacus Concepts, Cary, NC). The significance of differences between or among groups was examined using ANOVA followed by Tukey or Dunnett post-tests.
